# Wholesome Imperfection

**Published:** 1914-05

**Authors:** 


### Wholesome Imperfection.

Dr. A. Lawrence Lowell's article on the Danger to the Maintenance of High Standards from Excessive Formalism, in the **Journ. of the A. M. A.**, March 14, 1914, reminds us that the majority of leaders of medical thought and pleaders for advanced standards of education, have, themselves, been graduated from medical schools that would scarcely be admitted to Class C at present, provided that they maintained the same standards as in the past, with allowance for changes in actual knowledge.

Some knockers claim that the graduates from the medical college that comes the nearest to ideal standards in matriculation and graduation requirements, methods of pedagogy, equipment, etc., have yet to produce their first really great man. We do not altogether agree with this argument, but the literal statement is difficult to refute, and when we have pleaded that more time should be given, men have been named, within the same time limit, who have graduated from less perfect but perhaps more practical schools, and have actually attained deserved prominence.

One of the best incentives to success, in the best sense, is a humble realization both of personal imperfections and of lack in general and technical training. It ought to be self evident to an intelligent young man that all branches of learning are advancing, and that medicine is advancing rather more rapidly than the average science because it is still rather more hampered by definite lack of knowledge and a strong suspicion of wrong conceptions, than are most other sciences. The better trained a young man's brain has been, the better the medical school has ministered to his comfort, the easier and more objective it has made his acquisition of knowledge, the more perfectly it has trained him according to present standards, the more he ought to realize his duty to keep up the process. If, on the contrary, he is turned out with a complacent sense of his own perfection and superiority, he might better have entered a medical school with flaws in his spelling and grammar, an entire innocence of foreign languages, he might better have dissected a poorly prepared subject in a room with a frozen faucet, hustled around for obstetric and dispensary experience outside the school, and gone out into the world at the end of two short and inadequate sessions, with a strong sense of his unpreparedness.

